# *CRABP2* Promotes Myoblast Differentiation and Is Modulated by the Transcription Factors MyoD and Sp1 in C2C12 Cells

**DOI:** 10.1371/journal.pone.0055479

**Published:** 2013-01-31

**Authors:** Jing Yuan, Zhonglin Tang, Shulin Yang, Kui Li

**Affiliations:** 1 State Key Laboratory for Animal Nutrition, Institute of Animal Science, Chinese Academy of Agricultural Sciences, Beijing, People's Republic of China; 2 College of Animal Science, Yangtze University, Jingzhou, People's Republic of China; University of Cincinnati, United States of America

## Abstract

Cellular retinoic acid binding protein 2 (*CRABP*2), a member of a family of specific carrier proteins for Vitamin A, belongs to a family of small cytosolic lipid binding proteins. Our previous study suggested that *CRABP2* was involved in skeletal muscle development; however, the molecular function and regulatory mechanism of *CRABP*2 in myogenesis remained unclear. In this study, we found that the expression of the *CRABP2* gene was upregulated during C2C12 differentiation. An over-expression assay revealed that *CRABP2* promotes myogenic transformation by regulating the cell cycle during C2C12 differentiation. The region from −459 to −4 bp was identified as the core promoter and contains a TATA box, a GC box and binding sites for the transcription factors MyoD and Sp1. Over-expression, site-directed mutagenesis and EMSA assays indicated that the transcription factors MyoD and Sp1 regulate *CRABP*2 expression and promote myoblast differentiation in C2C12 cells.

## Introduction

Myogenesis can be regarded as a two-step process consisting of the determination, in which the satellite cells commit to the mature muscle lineage, and subsequent differentiation of mononuclear myoblasts to multinuclear myotubes [1]. A previous study documented that several myogenesis factors (MyoD, Myf5, MRF4 and MyoG as well as members of the MyoD gene family) are critical for the determination and terminal differentiation of skeletal muscle cells [2]. In cultured non-muscle cells, the exogenous expression of the MyoD gene family can induce myogenic differentiation [3], [4], [5], [6]. The MyoD gene family proteins bind to the CANNTG sequence (also known as the E-box sequence), which is present in the promoters and enhancers of many muscle-specific genes [7], [8]. Through the DNA-protein interaction, the MyoD gene family induces a conformational change and promotes muscle cell-specific gene transcription. Additionally, the MyoD gene family also promotes cell differentiation and inhibits the cell cycle [9].

Sp1 belongs to a family of zinc-finger transcription factors involved in the early development of an organism [10], [11]. It contains 3 zinc finger motifs, which bind to GC-rich sequences. Specifically, the Sp1 protein recognizes a motif of GGGCGG or other related GC-rich sequences [12]. The Sp1 protein regulates the expression of several genes involved in cell differentiation and embryonic development, enhancing or repressing gene activity [13], [14], [15], [16].

Vitamin A and its related molecules (retinol, retinaldehyde and retinoic acid) specifically bind many distinct cytoplasmic proteins and play essential roles in vision, growth, reproduction and cell differentiation [17]. The cellular retinoic acid binding proteins (CRABPs) are well-characterised members of a large family of small proteins that specifically bind retinoic acid [18] and mainly exert their biological function within cells. The CRABPs are abundantly expressed in numerous developing tissues [19], which suggests that CRABPs may perform specific functions during morphogenesis [20], [21]. *CRABP2* is a low molecular mass (15 kDa) protein that belongs to the multi-gene family of cellular retinoic acid binding proteins [22]. During mouse embryonic development, *CRABP2* was expressed in many organs [21], we used the GNF SymAtlas expression datasets ( http://symatlas.gnf.org/SymAtlas/), which demonstrated that *CRABP2* mRNA was upregulated from day 6.5 to day 10.5. These data indicate that the *CRABP2* gene may play a vital role during embryonic development.

The precise biological function and regulatory mechanism of the *CRABP*2 gene was unknown. Our recent LongSAGE analysis indicated that the *CRABP*2 gene contributes to prenatal skeletal muscle development in pigs [23]. Myoblast differentiation is a critical molecular event during foetal muscle development. We hypothesised that the *CRABP2* gene plays an important role in myogenesis. In this study, we analysed the biological function and regulatory mechanism of the *CRABP2* gene in C2C12 cells.

## Results

### The *CRABP2* gene was upregulated during C2C12 differentiation

**Figure 1:**
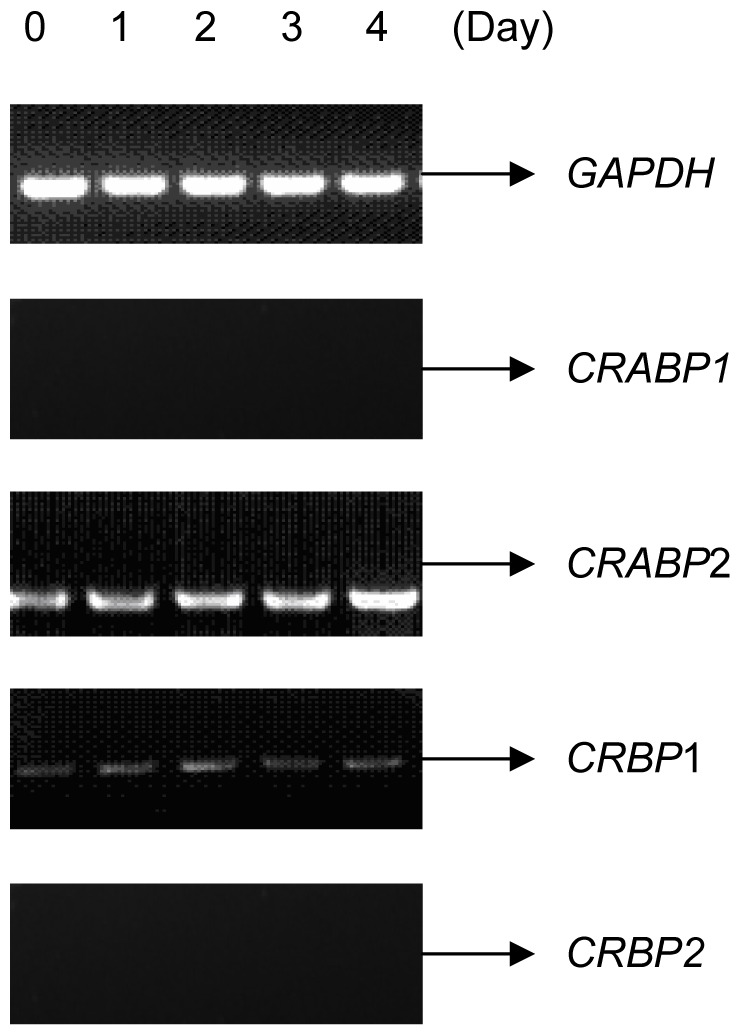
The expression of *CRABP1*, *CRABP2*, *CRBP1* and *CRBP2* genes according to RT-PCR analysis during C2C12 differentiation. 0: C2C12 myoblast cells; 1–4: Days 1–4 in C2C12 cell myoblasts differentiated into myotubes.

**Figure 2:**
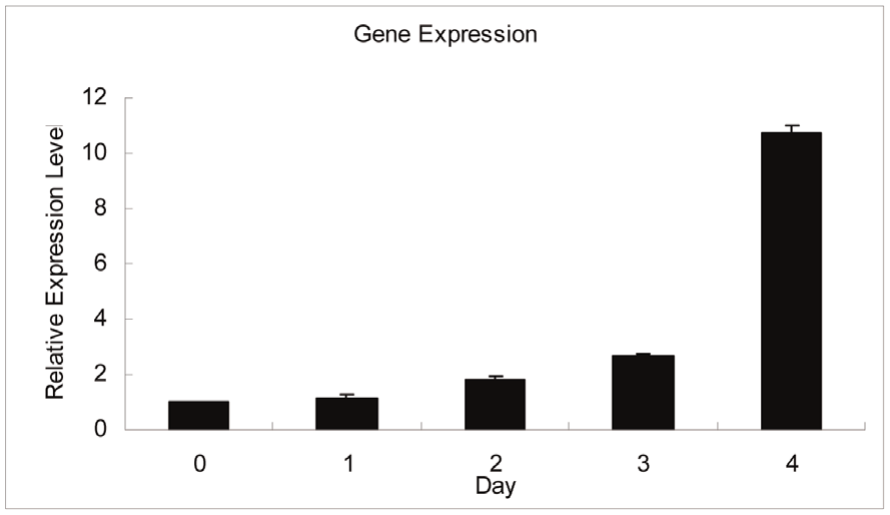
The expression of the *CRABP2* gene during differentiation was assessed by quantitative real-time PCR (qRT-PCR). The values were normalized to *GAPDH* mRNA expression level and the value of day 0 was set to 1. The error bars indicate the SD (n = 3).

We analysed the expression of the genes (*CRABP1*, *CRABP2*, *CRBP1*, *CRBP2*) expressing carrier proteins for Vitamin A and related molecules by RT-PCR during C2C12 differentiation. The results shown reveal expression levels of *CRABP1*, *CRABP2*, *CRBP1*, *CRBP2* observed. We found that the *CRABP1* and *CRBP2* genes were not detected and the mRNA expression of the *CRBP1* gene was unchanged, but the *CRABP2* gene was markedly upregulated (Figure 1). To further test the expression change of the *CRABP2* gene during C2C12 differentiation, quantitative real-time PCR (qRT-PCR) was performed. The results suggested that *CRABP2* was upregulated from day 0 to day 4 during myogenic differentiation in C2C12 cells (Figure 2).

### *CRABP2* promotes C2C12 differentiation

**Figure 3:**
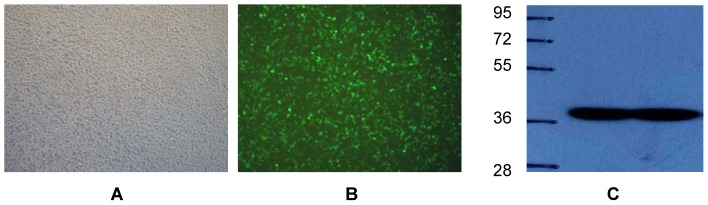
The cell morphology of 293T under fluorescence microscope (Olympus, micropublisher 3.3RTV, 100×) transfected by CRABP2 overexpression vector and the expression of CRABP2 detected by Westernblot. (A) Visible image transfected with lentivirus; (B) Green fluorescence image transfected with lentivirus; (C) CRABP2 expression by westernblot.

To study the effects of *CRABP2* in C2C12 cells, we constructed CRABP2 lentivirus vector for transit gene expression. Analysis by western blotting verified that *CRABP2* was expressed in 293T cells (Figure 3) and the titer of the lentivirus particle was 2E +9 TU/ml.

**Figure 4:**
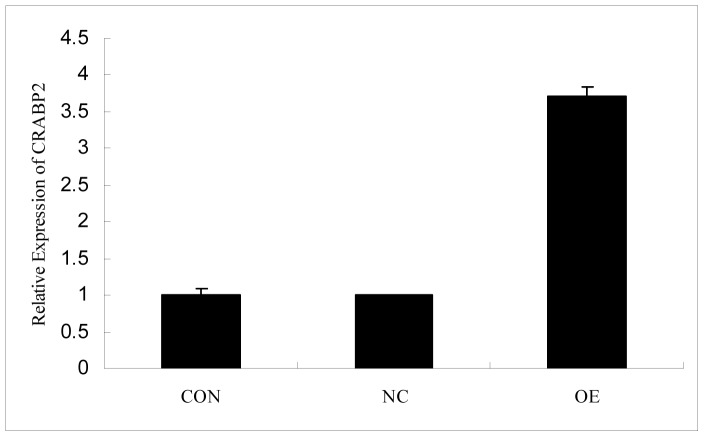
The target gene expression in C2C12 cells transfected with the *CRABP2* lentivirus vector. (CON: Blank control; NC: Negative control; OE: Overexpression).

**Figure 5:**
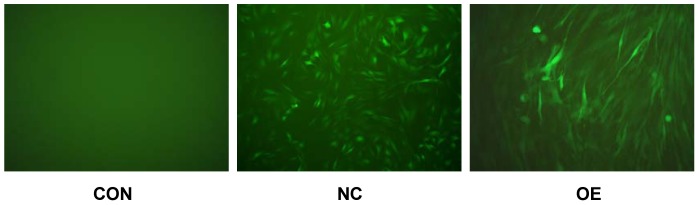
The cell morphology of C2C12 under fluorescence microscope (Olympus, micropublisher 3.3RTV, 200×) transfected by different lentivirus vector. (CON: Blank control; NC: Negative control; OE: Overexpression).

After transfecting C2C12 cells with CRABP2 expression vector, we observed cell morphology by fluorescence microscopy and studied a mixture of cell populations with various CRABP2 expression levels by qRT-PCR. We found that the expression levels of *CRABP2* significantly increased (Figure 4) and that the cell morphology was characteristic of differentiation (Figure 5) in over-expression (OE) group compared with Blank control (CON) and Negative control (NC) groups.

**Figure 6:**
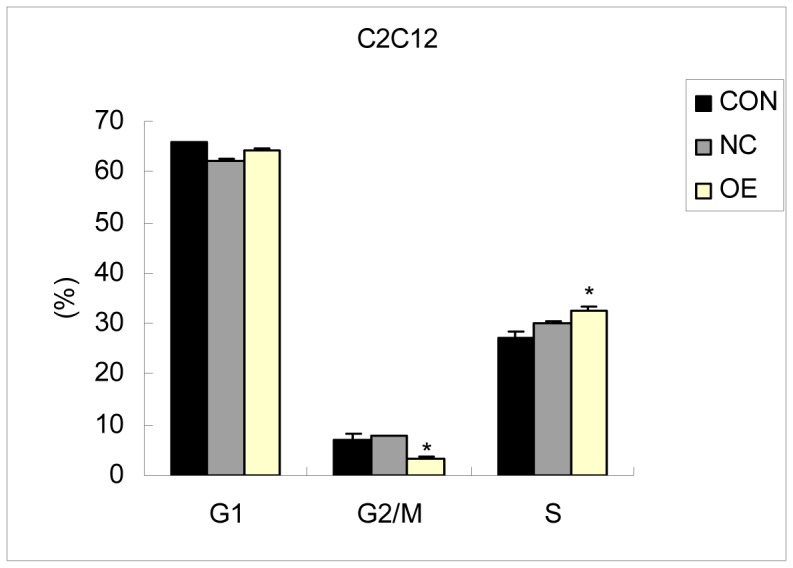
The FCM results of the C2C12 cells transfected with the *CRABP2* lentivirus vector. (CON: Blank control; NC: Negative control; OE: Over expression).

In contrast with the CON and NC groups, FACS cell sorting results of the OE group showed no significant changes in the percentage of cells in the G1 phase, but the percentage of cells in the G2/M phase decreased and the percentage of cells of S phase increased (*p*<0.05) (Figure 6). It has been reported that the number of cells in G2/M phase decreases and more cells remain in S phase during the early differentiation of C2C12 cells [24]. Because the percentage of S phase cells was higher and the percentage of G2/M phase cells was lower in OE group than control group, indicating that S phase arrested and the number of cells entered into G2 phase decreased. These data demonstrated that DNA synthesis reduced, C2C12 cell no longer continue to proliferate, may enter the cell differentiation.

### Identification of the core promoter region

**Figure 7:**
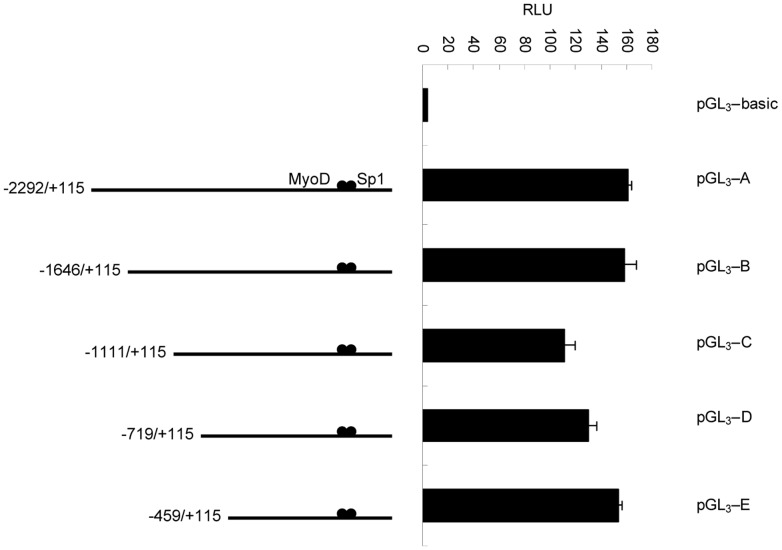
The first deletion analysis of the promoter of the mouse *CRABP2* gene in C2C12 cells. C2C12 cells were co-transfected with various promoter regions fused to firefly luciferase and a Renilla luciferase expression control vector. The resulting firefly luciferase activity was then normalized to Renilla luciferase activity, and the relative values were presented as the fold-increase over the activity of the promoter-less pGL_3_-basic vector. Values represent the mean ± SD of three independent experiments.

To determine the functional fragment of the *CRABP2* promoter, we inserted a 2.4 kb fragment into the promoter-less vector pGL_3_-basic. The resulting plasmid (pGL_3_-A) was transfected into C2C12 cells, and the luciferase activity measured. Compared with cells transfected with pGL_3_-basic, the cells transfected with the pGL_3_-A plasmid had significantly increased luciferase activity, revealing that the 2.4 kb fragment was a functional promoter fragment. To further identify the precise promoter region, the four promoter luciferase reporter plasmids (pGL_3_-B, pGL_3_-C, pGL_3_-D and pGL_3_-E) were constructed by further deletion using pMD18 T-A as a template. As shown by transient transfection results, the pGL_3_-E plasmid contained the basal promoter activity (Figure 7).

**Figure 8:**
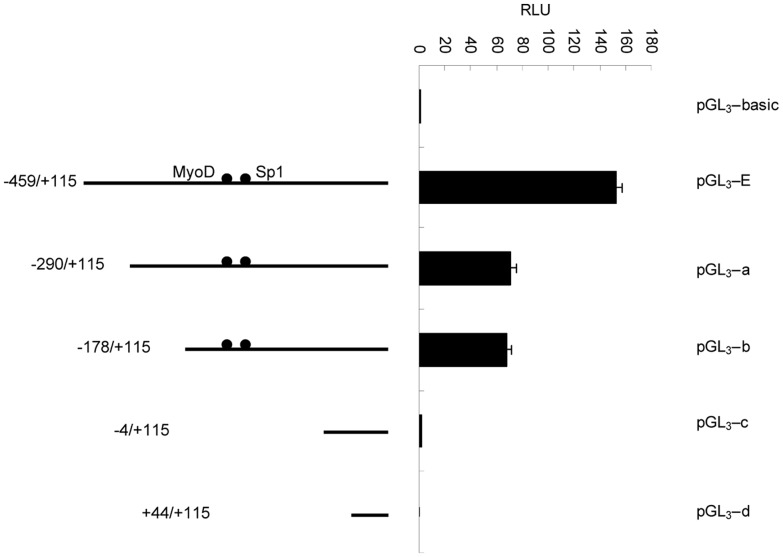
The second deletion analysis of the promoter of the mouse *CRABP2* gene in C2C12 cells. C2C12 cells were co-transfected with various promoter regions fused to firefly luciferase and a Renilla luciferase expression control vector. The resulting firefly luciferase activity was then normalized to Renilla luciferase activity, and the relative values were presented as the fold-increase over the activity of the promoter-less pGL_3_-basic vector. Values represent the mean ± SD of three independent experiments.

Four promoter luciferase reporter plasmids (pGL_3_-a, pGL_3_-b, pGL_3_-c and pGL_3_-d) were constructed by further deletion of pGL_3_-E. The results indicated that the pGL_3_-E plasmid with the −459 bp to −4 bp fragment contained the minimal core promoter region (Figure 8).

### Identification of *MyoD* and *Sp1* as transcription factors of the *CRABP2* gene

Bioinformatic analyses using TFSEARCH, TESS, Web Promoter Scan Service and MatInspector programs indicated that the *CRABP2* promoter contains a TATA box, a GC box and binding sites for the transcription factors MyoD and Sp1. The core promoter region contains the TATA box and the GC box, as well as MyoD and Sp1 binding sites.

**Figure 9:**
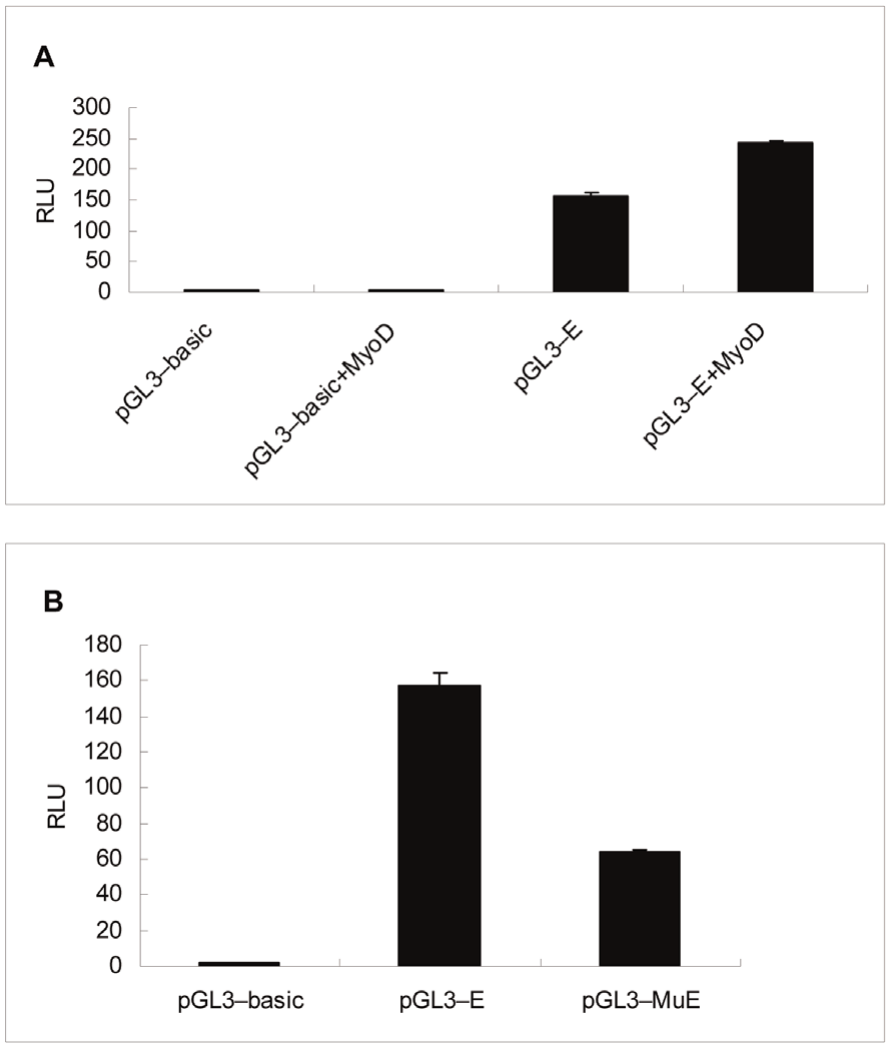
Transcriptional activation of the *CRABP*2 promoter was effected by *MyoD* and *Sp*1. (A) Transcriptional activation of the *CRABP2* promoter was potentiated by the MyoD expression plasmid. The empty vector or the core promoter plasmid pGL_3_-E was cotransfected with the MyoD expression plasmid pcDNA3.1-MyoD into C2C12 cells. The over-expression of MyoD increased the *CRABP2* promoter activity (n = 3); (B) The transcriptional activation of the *CRABP2* promoter was repressed by the Sp1 site-directed mutation vector. The Sp1 binding site was required for *CRABP2* promoter function, and the transcription factor Sp1 facilitated the transcriptional activation of the *CRABP2* gene. Values represent the mean ± SD of three independent experiments.

To determine experimentally that MyoD and Sp1 are regulators of the *CRABP*2 gene, an over-expression vector of MyoD and a site-directed mutation vector of Sp1 were constructed. Co-transfection with the minimal core promoter vector was performed to verify the transcription factors MyoD and Sp1, respectively. The results showed that the *MyoD* and *Sp*1 sites in the promoter region were necessary and functional to drive the basal reporter expression in transient transfection assays in C2C12 cells (Figure 9).

### Validation of MyoD and Sp1 transcription factors as regulators for the *CRABP2* gene

To further confirm the function of *MyoD* and *Sp*1, electrophoretic mobility shift assays (EMSA) were carried out to analyse binding capabilities in cell nuclear extracts.

**Figure 10:**
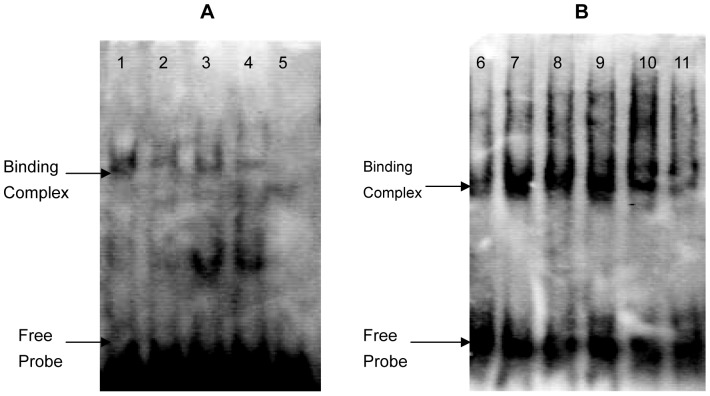
Binding activities of MyoD and Sp1 in nuclear extracts. (A) Assay of binding activity of MyoD in nuclear extracts; (B) Assay of binding activity of Sp1 in nuclear extracts. Lanes 1, 6: nuclear extracts from C2C12 myoblasts; lanes 2, 3, 4, 7, 8, 9: nuclear extracts from C2C12 myotubes after a 4 day induction with horse serum; lanes 5, 11: competition for binding by the unlabeled probe; lane 10: positive control.

Oligonucleotides were synthesized and BIO-labelled and then incubated with nuclear extracts from C2C12 cells. Specific DNA-protein complexes were identified from C2C12 nuclear extracts (Figure 10). The binding activities were different between C2C12 myoblasts and C2C12 myotubes. In C2C12 myotubes, the binding activity of MyoD was decreased; however, the binding activity of Sp1 was enhanced. These results indicate that MyoD and Sp1 specifically bind to promoter elements to regulate *CRABP*2 expression in C2C12 cells at different times during differentiation.

## Discussion

Our previous LongSAGE analysis suggested that *CRABP*s were involved in skeletal muscle development in the pig throughout embryonic development.^23^ Therefore, the aim of this study was to investigate the function of *CRABP*s during the process of differentiation of C2C12 cells into myotubes. The RT-PCR and real time PCR analyses suggested that *CRABP2* was increasingly expressed during C2C12 cell differentiation. Interestingly, we found that *CRABP*2 over-expression accelerated the process of C2C12 differentiation into myotubes in vitro. These observations suggest that *CRABP2* contributes to the process of C2C12 myoblast differentiation into myotubes.

We then carried out a promoter analysis through a step-by-step deletion process to identify the core promoter region. Bioinformatics analyses showed that the promoter region contained a TATA box, a GC box, and MyoD and Sp1 transcription factor binding sites. We constructed a MyoD over-expression vector and an Sp1 site-directed mutation vector. Co-transfection was performed to demonstrate the effects of MyoD and Sp1 transcription factors on the *CRABP2* gene. The EMSA results indicated that the predicted MyoD and Sp1 binding sites bound to the MyoD and Sp1 proteins, respectively, in nuclear extracts from C2C12 cells. Additionally, excess amounts of unlabelled oligonucleotides inhibited the binding activities in C2C12 cells. This result further proved that the MyoD and Sp1 binding sites were functional regulators for the *CRABP2* gene. These results demonstrated that the putative MyoD and Sp1 binding sites in the core promoter of *CRABP2* may be responsible for transcriptional regulation in C2C12 cells.

Transcription factors are trans-regulatory factors that exert biological function by interaction with cis-regulatory elements of structural genes. The MyoD family recognizes and binds the consensus sequence CANNTG (N for any nucleotide), and the promoter region of *CRABP2* contains a CANNTG sequence. The MyoD gene family forms a very complex regulatory network by interacting with a number of positive and negative factors to regulate *CRABP2* gene transcription, controlling cell growth and cell differentiation. The Sp1 transcription factor was involved in regulating cell growth and controlling morphogenetic pathways both in mammals and in invertebrates [25], [26]. In our study, Sp1 regulated gene transcription by binding the GGGCGG sequence in specific regulatory sites of the *CRABP2* gene. Therefore, MyoD and Sp1 are important regulators for the *CRABP2* gene and directly bind to the promoter region to influence *CRABP2* expression. *CRABP2* is in turn involved in regulating C2C12 cell differentiation.

In summary, *CRABP2* expression was upregulated from day 0 to day 4 at the mRNA level in differentiating C2C12 cells. The over-expression of *CRABP2* led to cell cycle changes in C2C12 cells in vitro. We have identified a biological function for *CRABP2* in C2C12 differentiation and found that MyoD and Sp1 play a regulatory role in *CRABP2* gene transcription by binding in the core promoter region between −459 bp and −4 bp upstream of the *CRABP2* gene. These results give new insight into the biological function and regulatory mechanism of the *CRABP2* gene.

## Materials and Methods

### Cell culture and RNA extraction

C2C12 cells were cultured in DMEM (Dulbecco's Modified Eagle's Medium) supplemented with 10% FBS, 2 mM glutamine, 100 units/ml penicillin and 100 µg/ml streptomycin and were maintained at 37°C in 5% CO_2_. The C2C12 cells were seeded in six-well plates. After approximately 12–16 h, when cell confluence reached approximately 60%–70%, the differentiation of C2C12 myoblasts into myotubes was induced by the addition of differentiation medium (DMEM containing 2% horse serum instead of 10% FBS).

Starting at the beginning of differentiation, the C2C12 cells were cultured in six-well plates and harvested for RNA extraction at 0, 1, 2, 3 and 4 days. Total RNA samples were extracted using TRIZOL (Invitrogen, USA) according to the manufacturer's instructions. The cDNA samples were obtained by reverse transcription from 1 µg RNA using oligo(dT) and M-MLV reverse transcriptase (Promega, USA).

### RT-PCR analysis

The RT-PCR (Reverse Transcription-Polymerase Chain Reaction) was performed in a volume of 10 µl containing 1 µl of 10× PCR buffer (plus Mg^2+^), 100 µmol/L of each dNTP, 0.5 µmol/L of each PCR primer, 1.0 U Taq DNA polymerase (Takara, Japan) and 0.5 µl of cDNA template. The primers were designed and synthesized as shown in Table 1. The PCR reaction was 5 min at 95°C followed by 27 cycles of 30 s at 95°C, 30 s at the specific melting temperatures of each primer pair, 30 s at 72°C and a final extension time of 5 min at 72°C.

**Table 1:** Primers used for gene expression analysis in the study.

Primer	Sequence (5′– 3′)	Tm (°C)	Products Length (bp)
C1F	GGTGTGAACGCCATGCTGAG	62°C	323 bp
C1R	GTGCACACCACATCATCGGC		
C2F	ACCTCCACCACTGTGCGAAC	58°C	293 bp
C2R	CGGAAGTCGTCTCAGGCAGT		
R1F	AGAGATCGTGCAGGATGGCG	58°C	383 bp
R1R	GGTGGGTATGCGTTTCGGTC		
R2F	GGCCACCATCATGACGAAGG	62°C	342 bp
R2R	ACCCACTGCTTCCAGCCACG		
R4F	TCCGTCTTCTGAGCAACTGG	62°C	351 bp
R4R	GCCTGCTTTGACAGTAACCA		
R7F	TCTTCTCAGCAGCGACAACT	62°C	377 bp
R7R	ATCAGGCTCTCTGGAAGGTT		
C2-GF	ACTCAGCGTCCAGTGTTCTAG	61°C	512 bp
C2-GR	CGGAAGTCGTCTCAGGCAGT		
C2-AI-F	TCGCCACCATGCCTAACTTTTCTGGCAAC	55°C	460 bp
C2-AI-R	CTCTCGGACGTAGACCCTG		
MyoD-GF	GACAGGGAGGAGGGGTAGAG	61.4°C	1166 bp
MyoD-GR	GAAGAACCAGGGACACCATC		
GAPDH-F	GGTGAAGGTCGGTGTGAACG	60°C	233 bp
GAPDH-R	CTCGCTCCTGGAAGATGGTG		

C1F, C1R; C2F, C2R; R1F, R1R; R2F, R2R; R4F, R4R; R7F, R7R: primers used for determining the expression of *CRABP1*, *CRABP2*, *CRBP1*, *CRBP2*, *RBP4*, *RBP7* during C2C12 differentiation time; C2-GF, C2-GR and C2-AI-F, C2-AI-R: primers used for construction of the *CRABP2* over-expression vector; MyoD-GF, MyoD-GR: primers used for construction of the MyoD over-expression vector; GAPDH-F, GAPDH-R: primers used as an internal control.

### Real-time PCR analysis

Real-time PCR amplifications were carried out using the Light Cycler Real Time PCR instrument (model 7500; Applied Biosystems, Inc., Foster City, CA). The PCR reaction contained 50× ROX Reference DyeII, 2× SYBR Green Realtime PCR Master Mix (Takara, Japan), 10 µM primers and 1.0 µl of cDNA template. Pure water was added for a total volume of 20.0 µl. Gene amplification was performed under the following conditions: 95°C for 30 s, followed by 40 cycles of 95°C for 5 s, 60°C for 20 s and 72°C for 34 s. The PCR was performed in biological triplicate for each condition. The target gene data was analysed and normalized to *GAPDH* mRNA levels.

### Construction and transfection of the *CRABP2* lentivirus vector

The vector overexpressing *CRABP2* was constructed to study its function in C2C12 cells. The cDNA fragment was isolated and ligated into the pGC-FU vector linearized by cutting with Age I restriction enzyme, purchased from GeneChem Co., Ltd, Shanghai, China. The expression plasmid containing the fusion protein was extracted using ultrapure endotoxin-free extraction kits (Omega, USA). The transfection of 293T cells was carried out using Lipofectamine 2000 according to the manufacturer's instructions. After a 48 h incubation, the cell supernatant containing virus-like particles was collected. After concentration, the viral titres were determined in the 293T cells.

The C2C12 cells were plated on 6-well plates and cultured overnight at 37°C in 5% CO_2_. When cell confluence reached approximately 60–70%, lentivirus of a specific quantity was added to cells. After 12 h, the transfection medium was removed and replaced with normal growth medium.

After 3–4 days, the GFP expression was tested by fluorescence microscopy to determine transfection efficiency. The cells were then harvested and used for the cell cycle assay and RNA extraction. All transfections were performed in triplicate for each plasmid.

### Flow cytometry analysis (FCM)

The C2C12 cells were collected and centrifuged at 1200 rpm for 5 min. The cells were washed twice in 4°C pre-chilled PBS (phosphate buffered saline, pH = 7.2–7.4), fixed with 4°C pre-chilled 70% ethanol, centrifuged at 1500 rpm for 5 min to remove stationary phase cells and resuspended in PBS. The cells were filtered once through 400 mesh and centrifuged at 1200 rpm for 5 min, and after the PBS supernatant was removed, the cells were then stained with 1 ml of PI at 4°C in darkness for 30 min. Finally, the cells were analysed by flow cytometry (FACSCalibur, BD, USA).

### Construction of the luciferase reporter plasmids

The 5′-upstream 2.4 kb promoter fragment of the *CRABP2* gene was amplified from mouse genomic DNA and then inserted into the pMD18-T vector. It was named pMD18 T-A.

The construction of the luciferase reporter gene plasmid was carried out as follows: the pMD18 T-A vector was cut using MluI and NcoI restriction enzymes and then ligated into linearized basic vector (named pGL_3_-A). Fragments of different lengths from the 5′-upstream sequence were amplified using pMD18T-A as a template and then ligated into linearized pGL_3_-basic vector (named pGL_3_-B, pGL_3_-C, pGL_3_-D and pGL_3_-E). The plasmids pGL_3_-A, pGL_3_-B, pGL_3_-C, pGL_3_-D and pGL_3_-E were constructed to contain −2292 bp to +115 bp, −1646 bp to +115 bp, −1111 bp to +115 bp, −719 bp to +115 bp and −459 bp to +115 bp of the *CRABP2* gene, respectively.

To identify more precisely the core promoter region, we performed further deletions on the promoter fragment pGL_3_-E: four luciferase reporter gene plasmids, pGL_3_-a (−290 bp to +115 bp), pGL_3_-b (−178 bp to +115 bp), pGL_3_-c (−4 bp to +115 bp) and pGL_3_-d (+44 bp to +115 bp), were constructed using the above methods. Each promoter luciferase plasmid was constructed using a sequence-specific forward primer and a common reverse primer (shown in Table 2). The sequences were analysed by Invitrogen.

**Table 2:** Primers used to make constructs to analyse promoter activity in the study.

Primer	Sequence (5′– 3′)	Tm (°C)	Products Length (bp)
C2-AF	GCTGCTCATGTCTGCGATTC	61°C	2408 bp
C2-BF	AACTGAAAATCATCCCGTTGTG	61°C	1762 bp
C2-CF	AGGCAGGGACCTTGTTTGAGAT	61°C	1227 bp
C2-DF	GGGAAGCGTAGCCACCGA	61°C	833 bp
C2-EF	CTGTTTCATTGAGTCCATTTCG	61°C	575 bp
C2-aF	CGAGAGAGCATCCGTGACCC	61°C	406 bp
C2-bF	TATCTTGGCTCTTGGAGAACCC	61°C	294 bp
C2-cF	CCCAGCGGCTGTGCAATT	61°C	120 bp
C2-dF	AGGATCTGTTCTGCAAAGGAGA	61°C	72 bp
C2-R	TCAACTAGAACACTGGACGCTG		

C2-A, C2-B, C2-C, C2-D, C2-E, C2-a, C2-b, C2-c and C2-d: Forward primers for construction of the luciferase reporter gene vectors pGL_3_-A, pGL_3_-B, pGL_3_-C, pGL_3_-D, pGL_3_-E, pGL_3_-a, pGL_3_-b, pGL_3_-c and pGL_3_-d, respectively; C2-R: Reverse primer for construction of luciferase reporter gene vectors pGL_3_-A, pGL_3_-B, pGL_3_-C, pGL_3_-D, pGL_3_-E, pGL_3_-a, pGL_3_-b, pGL_3_-c and pGL_3_-d.

### Bioinformatic and statistical analyses

The sequence of the 5′-upstream 2.4 kb promoter fragment of the *CRABP*2 gene was analysed using Promoter Scan (http://www.bimas.cit.nih.gov/molbio/proscan). The core promoter region was identified by the Transcription Element Search System (http://www.cbil.upenn.edu/cgi-bin/tess/tess), the Berkeley Drosophila Genome Project programs (http://www.fruitfly.org/seq_tools/promoter.html) and TFSEARCH (http://www.cbrc.jp/research/db/TFSEARCH.html).

All experiments were performed in triplicate, and the data were presented as mean ± SD. Student's t-test was used to determine statistical significance.

### Luciferase assay

The C2C12 cells were plated on 24-well plates and cultured overnight at 37°C to ensure approximately 60%-70% confluence. Co-transfections were performed using 2.0 µl of Lipofectamine 2000 reagent (Invitrogen, USA), 0.8 µg of the firefly luciferase plasmid DNA and 0.008 µg of pRL-TK plasmid DNA (Promega, USA) as an internal control. The transfection medium was removed and replaced with growth medium after 5 h.

The Firefly and Renilla luciferase activities were measured at 24 h after transient transfection using the Dual-Glo Luciferase assay system (Promega, USA) and a TD20/20 luminometer (Turner Designs) according to the manufacturer's instructions. Each plasmid was tested in three independent experiments. The luciferase activity was normalized using the Renilla luciferase activity levels and expressed as relative luciferase unites (RLU) to reflect the gene promoter activity.

### Construction of the MyoD expression vector

The cDNA sequence of the *MyoD* gene was amplified using mouse muscle mRNA with the primers listed in Table 1. The PCR product was cloned into the pMD18-T vector (Invitrogen, USA) and then inserted into the EcoRV/NotI sites in the linearized pcDNA3.1(+) vector (Invitrogen, USA). The recombinant plasmid was sequenced by Invitrogen.

### Construction of the Sp1 site-directed mutation vector

To identify Sp1 as a regulatory factor for the *CRABP2* gene in C2C12 cells, we constructed a site-directed mutation vector of Sp1. The site-directed mutagenesis of the Sp1 binding site was obtained by a two-step PCR protocol using pMD18 T-A as a template. The original GGC site was mutated to TTA. The mutagenic primers were 5′ AGGTGGTGTGGAAGGCGGTTAGGGGGCGGGGCCGCCTCATGCACCAGCT 3′ (forward primer) and 5′ CCTAACCGCCTTCCACACCACCT 3′ (reverse primer). Thermocycler parameters were set at 95°C for 5 min followed by 27 cycles of 95°C for 30 s, 60°C for 30 s and 72°C for 30 s with a final extension time of 5 min at 72°C. The final PCR product was cloned into pGL_3_-basic and named pGL_3_-muE.

### Electrophoretic mobility shift assay (EMSA)

Nuclear extracts from C2C12 myoblasts and myotubes were prepared according to the instruction manual of the NProtein Extraction kit (Exprogen, Beijing, China). The proteins were measured using a BCA Protein Assay Kit (Exprogen, Beijing, China). The probes for MyoD and Sp1 were synthesized by Integrated DNA Technologies (IDT, USA).

The binding reactions were performed in a 10 µl mixture containing 1.0 µl of 10× Binding Buffer, 1.0 µl of Poly (dI∶dC), 2 µg of nuclear extract and 0.5 µl of Bio-labelled probes. For the competition experiments, unlabelled probes were added to the binding reaction mixture and co-incubated. Subsequently, DNA-protein complexes were separated by 6.5% non-denaturing polyacrylamide gel electrophoresis in Tris-Boric acid (TBE) buffer. Electroblotting and chemiluminescence detection were performed according to the instructions of the BiotinLight™ EMSA Kit (Exprogen, Beijing, China). Results were observed by chemiluminescence imager.
